# Evidence of Many-Body Interactions in the Virial Coefficients of Polyelectrolyte Gels

**DOI:** 10.3390/gels8020096

**Published:** 2022-02-04

**Authors:** Ferenc Horkay, Jack F. Douglas

**Affiliations:** 1 Section on Quantitative Imaging and Tissue Sciences, Eunice Kennedy Shriver National Institute of Child Health and Human Development, National Institutes of Health, Bethesda, MD 20892, USA; 2 Material Measurement Laboratory, Materials Science and Engineering Division, National Institute of Standards and Technology, Gaithersburg, MD 20899, USA

**Keywords:** polyelectrolyte gel, phase separation, salt, multivalent ions, osmotic swelling pressure, osmotic compressibility, small angle neutron scattering, osmotic virial coefficient, swelling-deswelling transition

## Abstract

Simulation studies of aqueous polymer solutions, and heuristic arguments by De Gennes for aqueous polyethylene oxide polymer solutions, have suggested that many-body interactions can give rise to the ‘anomalous’ situation in which the second osmotic virial coefficient is positive, while the third virial coefficient is negative. This phenomenon was later confirmed in analytic calculations of the phase behavior and the osmotic pressure of complex fluids exhibiting cooperative self-assembly into extended dynamic polymeric structures by Dudowicz et al. In the present study, we experimentally confirm the occurrence of this osmotic virial sign inversion phenomenon for several highly charged model polyelectrolyte gels (poly(acrylic acid), poly(styrene sulfonate), DNA, hyaluronic acid), where the virial coefficients are deduced from osmotic pressure measurements. Our observations qualitatively accord with experimental and simulation studies indicating that polyelectrolyte materials exhibit supramolecular assembly in solution, another symptomatic property of fluids exhibiting many-body interactions. We also find that the inversion in the variation of the second (*A*_2_) and third (*A*_2_) virial coefficients upon approach to phase separation does not occur in uncharged poly(vinyl acetate) gels. Finally, we briefly discuss the estimation of the osmotic compressibility of swollen polyelectrolyte gels from neutron scattering measurements as an alternative to direct, time-consuming and meticulous osmotic pressure measurements. We conclude by summarizing some general trends and suggesting future research directions of natural and synthetic polyelectrolyte hydrogels.

## 1. Introduction

The phase behavior of materials ranging from gases to polymer solutions and gels, can often be understood semi-quantitatively in terms of the first few virial coefficients quantifying intermolecular interaction strength in the limit of low concentration [1,2,3,4,5]. The second (*A*_2_) and third (*A*_3_) virial coefficients, or their equivalent *χ_i_* -interaction parameters in a polymer solution context [6,7], have correspondingly been adopted as measures of intermolecular interactions having fundamental significance for materials classification, design, and characterization [8]. It is often implicit in this type of description of phase behavior that the interactions between the molecules can be described by a single pairwise decomposable interactions such as well-known Lennard-Jones or square-well potentials. However, it is not clear if patterns of phase behavior based on this type of ‘simple fluid’ apply to ‘complex liquids’, such as ionic and polyelectrolyte solutions, polyelectrolyte gels, etc. In these systems, many-body interactions associated with polymer and ion solvation and hydrogen bonding of the water molecules and the ‘hydrophobic effect’ governing the solvation of uncharged molecules or moieties in water that might lead to entirely distinct patterns of phase behavior. Along these lines, simulations of simple hydrophobic solutes (modeled as Lennard-Jones molecules or relatively simple molecules such as methane) in water at ambient temperatures have indicated that while the interaction of a few particles may be predominantly repulsive, the interactions among many particles become attractive when the number of molecules becomes sufficiently large [9,10,11]. Although computational studies differ in details, there is a broad consensus that *A*_2_ for model ‘hydrophobic’ solutes tends to be small in magnitude [12,13] so that the sign of *A*_2_ does not explain the general tendency of hydrophobic materials to macroscopically phase separate. For ‘simple’ fluids, many-body interactions are not prevalent, and the onset of phase separation can be understood by simply truncating the virial expansion describing the linear term in the pressure. In this approximation, we may roughly understand the onset of phase separation by the condition at which the osmotic pressure becomes negative, corresponding to a leading order virial expansion approximation to the condition, i.e., *A*_2_ *φ* ≈ −1, where *φ* is the volume fraction of the dissolved molecules, and *A*_2_ is described in dimensionless units defined below. In systems with appreciable many-body interactions, the third and higher virial coefficients can account for the attractive interactions exhibited by many interacting particles. De Gennes [14] recognized that a phenomenon of this kind seemed to be operative in understanding the phase behavior of polyethylene oxide in water. In particular, he simply assumed that *A*_2_ remained positive, corresponding to a repulsive binary polymer interchain interactions, and *A*_3_ and higher virial coefficients were negative, accounting for the attractive interchain interactions responsible for phase separation in this, and presumably other, water soluble polymers [14,15]. Notably, De Gennes introduced this physical model as a hypothesis intended to explain observations rather than as a result of any direct theoretical study.

In the polymer science field, the virials are often specified by Flory χ-parameters derived from the semi-empirical Flory-Huggins there where the polymer architecture is highly idealized. The virials involve no model assumptions and are related to the Taylor series expansion of the pressure and osmotic pressure, respectively, in non-ideal gases and solutions as a function of concentration. Even though these measures of interaction are defined in the dilute limit, these parameters play a significant role in describing thermodynamic properties of gases and liquid mixtures at essentially any concentration. The importance of these virials in characterizing solvent quality of polymer solutions and the phase stability of polymer blends and solutions is generally appreciated in polymer science, but it is not so generally appreciated that these interaction parameters provide a general metric for defining the complexity of fluids in terms of their equation of state scaling of thermodynamic properties.

Recent studies of many-body interaction effects, especially computational studies in the context of attempts at understanding the dynamics and thermodynamics of aqueous solutions, have provided significant insights into the influence of many-body interactions of various types on the virial coefficients. Even the simple case of water molecules in the gas phase is highly instructive. Kofke and coworkers [16,17] investigated the influence of the molecular polarizability, an obvious many-body effect not accounted for by standard pair potential models of water, and found that inclusion of this many-body contribution into the description of the intermolecular interaction gave rise a substantial improvement in the agreement between *A*_2_ and *A*_3_ data over a wide temperature range. The change in *A*_3_ was especially large in magnitude, and the many-body interactions associated with molecular polarizability could even change the sign of *A*_3_ for some temperatures. Later calculations showed that three-body effects were essential in understanding the third virial coefficients associated with CO_2_ dissolved in water [18,19,20]. Full quantum calculations of the second and third virial coefficients of water based on ab initio potentials have further confirmed the crucial dependence of three-body potentials in understanding the temperature variation of the second and third osmotic virial coefficients [21], confirming the conclusions of earlier work of Kofke and coworkers based on classical molecular dynamics methods and semi-empirical approximations of the many-body polarizability interactions. Recently, there have been many advances in modeling many-body interactions in aqueous solutions [22,23] and other forms of condensed matter.

More limited progress has been made in relation to developing an analytic theory that can address these many-body effects on the virial coefficients. Almost all existing analytic theories and even numerical calculations, apart from the limited cases noted above, have been for pairwise decomposable interaction potentials and our understanding of the phase behavior of liquids is largely based on this type of model, even though many-body interactions arise in many material systems. It is characteristic of many systems exhibiting many-body interactions to show particle clustering and it was exactly this tendency of polyethylene oxide (PEO) molecules to reversibly associate in solution that motivated De Gennes’ ‘cluster’ model mentioned above in which *A*_2_ was assumed to be positive and the phase separation of PEO in water was assumed to arise from attractive many-body interactions associated with many-chain associations. No specific statistical mechanical model was mentioned, although Bekiranov et al. [15] tried to construct a model that preserved the spirit of De Gennes’ arguments. Dudowicz et al. [24] later derived a model of the phase behavior of supramolecularly associating molecules in solution, which exactly gave rise to the pattern of phase behavior postulated by De Gennes. In this analytic model, the second virial coefficient of molecules exhibiting moderately attractive pairwise interactions possess a relatively slowly varying *A*_2_ with temperature over a large tempearture range, and the onset of phase separation upon cooling is triggered by *A*_3_ changing sign before *A*_2_ [7]. This unusual phase behavior arises from many-body attractions associated with the cooperative assembly process and provides a highly instructive model for how the phase behavior can be modified by molecular self-assembly. Douglas et al. [7] attempted to develop a generalized corresponding states based on this model, taking it as being representative of many complex fluids, extending Pitzer’s analysis [25,26] for ‘simple’ fluids whose phase behavior, or at least critical temperature can be largely understood based on the ‘shape’ of the pair potential and associated *A*_2_. De Bruyn and Goldstein have correspondingly emphasized the general importance of three-body interactions in condensed liquids and the potential influence of these interactions on the effective *A*_2_ [27]. These many body effects have demonstrated importance in complex fluids in polymer blends and solutions [7,28,29], where there are complex many-body effects associated with the shape dissimilarity of the monomer species and chain connectivity can be formulated minimally in terms of an equation of state involving *A*_2_, describing effective pairwise intermolecular interactions, and *A*_3_ describing the residual collective effect of the remaining many-body interactions.

Light and neutron scattering observations [30] and simulations [31,32] on highly charged natural and synthetic polyelectrolyte solutions have often indicated a propensity for dynamic large scale molecular clustering of polymer chains in solution, as evidenced by an ubiquitous large low-angle scattering intensity of polyelectrolyte solutions in the absence of a large amount of salt [30,31,32]. Although the clustering in these systems is not obviously equivalent to the chain-like assembly studied by Douglas and coworkers [7,24], it was suggested by them that the inverted variation of *A*_2_ and *A*_3_ in relation to phase instability should be a general signature of complex fluids exhibiting strong many interaction effects. The present work checks this hypothesis by examining *A*_2_ and *A*_3_ for a range of highly charged polyelectrolyte gels: sodium polyacrylate gels [33,34,35], DNA gels [36], hyaluronic acid gels [37], sulfonated polystyrene gels and model neutral polyvinyl acetate gels, providing some contrast in our comparison with simple phase behavior in fluids in which many-body interactions are not as strong. The polyelectrolyte gels considered here were initially in the form of the Na^+^ salt of the polymer before swelling. As well known, the addition of a Ca salt decreases the ‘solubility’ of charged polyelectrolyte gels and phase separation will be triggered at a critical Ca salt concentration. In this context, we study how *A*_2_ and *A*_3_ vary as the salt concentration is varied. As we anticipated, we find that the phase separation in each polyelectrolyte gel is signaled by a relatively sharp drop in the magnitude of *A*_3_, followed by a change of sign that signals the onset of phase separation. *A*_2_, on the other hand, exhibits little variation and remains positive even as the polyelectrolyte gel exhibits phase separation. This pattern of phase behavior, exhibited by all the polyelectrolytes that we studied, is contrasted by neutral gels in which *A*_2_ exhibits an appreciable change with temperature, but *A*_3_ remains relatively constant. This is typical behavior for both non-ideal gases and polymer solutions [7]. We thus observe clear evidence of many-body interactions in polyelectrolyte solutions. This pattern of behavior is probably common in complex fluids exhibiting cooperative supramolecular assembly.

The present work is organized as follows. After highlighting the significance of the virial coefficients in the context of many-body interactions of polymer solutions and gels in the Introduction, we briefly discuss the relevant theoretical considerations needed to interpret osmotic and scattering observations made on polymer gels. Then, we report osmotic swelling pressure measurements for typical synthetic and biopolymer gels. These gels differ in chain flexibility, backbone hydrophilicity, and cross-linking procedures (DNA and hyaluronic acid gels were made by cross-linking polymer chains in solution while poly(acrylic acid) (PAA) and sulfonated polystyrene (PSS) gels by cross-linking polymerization).

The osmotic observations made on polyelectrolyte gels are contrasted to measurements performed on neutral poly(vinyl acetate) gels swollen in isopropyl alcohol. This is followed by the analysis of the small angle neutron scattering (SANS) profiles of polyacrylic acid (PAA) and DNA gels. It is demonstrated that the dynamic component of the SANS intensity arising from thermodynamic concentration fluctuations is in reasonable agreement with the intensity estimated from macroscopic osmotic swelling pressure and shear modulus measurements. Finally, the Conclusion section summarizes the main results and we then describe some Future Directions in a final section.

## 2. Results and Discussion

### 2.1. Virial Coefficients (A_2_ and A_3_) of Polyelectrolyte and Neutral Polymer Gels

The osmotic properties of polymer solutions and gels are governed by the interaction between the polymer molecules and the solvent. Osmometry is a precise method that provides information about the phase stability of the gel or polymer solution and about the intermolecular interaction strength. Details about the osmotic pressure measurements are described in a separate section at the end of our paper.

In an osmotic swelling experiment, the derivatives of the free energy components are measured, i.e.,

*Π_tot_* = −*∂*(Δ*F_tot_*/*V*_1_)/*∂n*_1_ = *Π_elast_* + *Π_mix_* + *Π_ion_* (1)

where *Π_tot_* is the swelling pressure of the gel, *Π_elast_ Π_mix_* and *Π_ion_* are, respectively, the elastic, mixing and ionic contributions of *Π_tot_*, *V*_1_ is the molar volume of the solvent, and *n*_1_ is the number of moles of solvent. It is generally assumed that the elastic, mixing and ionic contributions are additive [6].

For networks made of flexible polymer chains the elastic contribution can be estimated from the classical theory of rubber elasticity, [38]

*Π_elast_* = −A *RT ν φ*^1/3^ = −G*_s_* (2)

where *ν* is the concentration of the elastic chains, *G_s_* is the shear modulus, *φ* is the volume fraction of the polymer, *R* is the gas constant, and *T* is absolute temperature. The constant A depends on the functionality of the network junctions. The osmotic mixing pressure *Π_mix_* can be given by the virial expression,

*Π_mix_* = − (*R*T/*V*_1_) [*ln* (1 − *φ*) + *φ* + *χ*_0_
*φ*^2^ + *χ*_1_
*φ*^3^ + …] = (*RT/V*_1_) (*A*_2_
*φ*^2^ + *A*_3_
*φ*^3^ + … ) (3)

where *A*_2_ and *A*_3_ are the second and third virial coefficients and *χ*_0_ and *χ*_1_ are the Flory interaction parameters.

### 2.2. Estimation of Π_elast_

**Figure 1:**
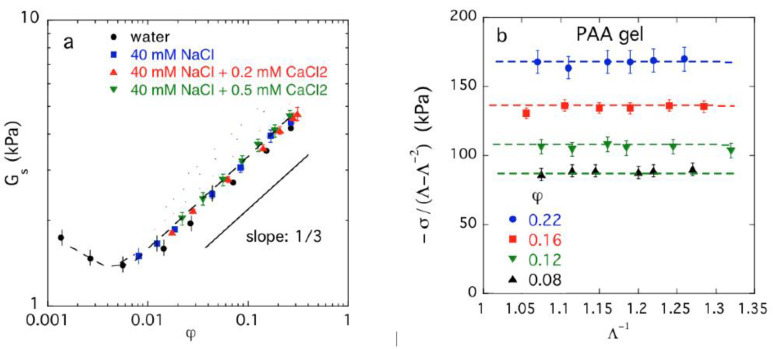
(**a**) Dependence of the shear modulus *G_s_* on the polymer volume fraction for PAA gels swollen in water and salt solutions. (**b**) Variation of *σ*/(*Λ* − *Λ*^−2^) with *Λ*^−1^ at different polymer volume fractions in PAA gels swollen in 40 mM NaCl solution with 0.2 mM and 0.5 mM CaCl_2_. In this figure *σ* is the nominal stress and *Λ* (= *L/L_o_*), where *L* and *L_o_* are the lengths of the deformed and undeformed gel specimen, respectively) is the deformation ratio. Dashed lines are guide to the eyes. The uncertainties are the standard deviation estimated from five repeated measurements. We note that we use the concentration unit mM, which is a shorthand notation for mmol/L. This notation for concentration is used for notational economy below.

Although the swelling dependence of the shear modulus of rubbery materials is generally a non-trivial problem, especially when the network material is swollen from the dry state [39], the classical network elasticity theory usually performs quite well for hydrogel materials, including polyelectrolyte hydrogels. In Figure 1a, we show a representative plot for PAA hydrogels swollen in different salt solutions, where the shear modulus is derived from the relation, *G_s_* ≡ *σ*/(*Λ* − *Λ*^−2^) (where *Λ* is the deformation ratio, see also caption of Figure 1b and section Materials and Methods). Deviations from the classical theory are only observed at small values of *φ*, corresponding to a large degree of network swelling where chain finite extensibility starts to become an issue. All our elasticity data for the hydrogels investigated in the present work, both charged and neutral gels, conform rather well to the classical network elasticity theory.

Figure 1 shows the effect of the divalent ion Ca^++^ on the elastic modulus of PAA gels. The influence of the counter-ion valence on the flexibility of polyelectrolyte chains, and on the overall network elasticity, raise questions about whether higher valence ions might lead to effective physical cross-links that would alter the elasticity of the hydrogels. To address this possibility, Horkay and coworkers [33,34,40,41] surveyed the effect of a range of monovalent (Li^+^, Na^+^, K^+^, Cs^+^) and divalent ions (Ca^++^, Sr^+2^, Ba^+2^) on the elasticity of model PAA, and other model polyelectrolyte gels, and found that these ions make no detectable effect on the elasticity of these networks. Evidently, the physical cross-linking hypothesis has no basis in any of the polyelectrolyte hydrogel materials that we investigate. Caution should then be given to invoking the idea that higher valence ions give rise to physical cross-links. Charge valence certainly influences *Π_mix_* in a fashion that only depends on ion valence for this surveyed family of metal counter-ions. Given this circumstance, we confine our discussion to monovalent Na^+^ and divalent Ca^++^ counter-ions and salts.

Although the occurrence of a direct attractive interaction between the polyelectrolyte chains mediated by metal ions can be excluded, there is clear experimental [42,43] and simulation evidence [44] that higher valence ions make polyelectrolyte chains appreciably more flexible under low salt conditions. Circumstantial evidence indicates that the higher valence counter-ions are more effective at screening the polymer-polymer charge interactions, which perhaps explains the diminished tendency towards supramolecular assembly in comparison with monovalent salt polyelectrolyte solutions [30]. We discuss evidence below for the general occurrence of supramolecular assembly in both polyelectrolyte solutions and polyelectrolyte gels based on neutron scattering measurements.

### 2.3. Estimation of Π_ion_

The ionic contribution is due to the difference in mobile ion concentrations inside and outside the polyelectrolyte gel, which gives rise to an osmotic pressure difference, *Π_ion_*, between the gel and the equilibrium solution. According to the Donnan theory [6], *Π_ion_* can be estimated as,

(4) $$\Pi_{\mathrm{ion}}\;=\;RT\;\overset{N}{\underset{j=1}{\sum }}\;({c_{j}}^{\mathrm{gel}}-{c_{j}}^{\mathrm{sol}})$$

where *c_j_^gel^* and *c_j_^sol^* represent the concentrations of the ions in the gel and in the equilibrium solution, and *N* is the number of mobile ions in the system. Recent experimental studies, as well as molecular dynamics simulations [45] have indicated that in the presence of added salt the Donnan contribution is very small, i.e., the first two terms of Equation (1) provides a satisfactory description of the osmotic behavior of polyelectrolyte gels. The simulations showed that under equilibrium conditions approximate cancellation arises between the electrostatic contribution and the counter-ion excluded-volume contribution to the osmotic pressure. Based on these results, it was shown that the Flory–Huggins model for nonionic polymer solutions, which accounts for neither electrostatic effects nor counter-ion excluded-volume effects, fits both experimental and simulated data for polyelectrolyte solutions.

It has been reported that the effect of monovalent ions on the total swelling pressure of highly swollen polyelectrolyte gels is adequately described by the Donnan theory [33,34,40,41]. However, when divalent counter-ions are added to the equilibrium solution, the swelling degree is drastically reduced, and the Donnan theory fails to predict swelling behavior. Shear modulus measurements made on gels at different Ca^++^ concentrations do not indicate a significant change in the effective cross-link density (see Figure 1a). Therefore, the formation of stable bridges between the network chains with increasing Ca^++^ concentration can be ruled out. There is, however, a strong interaction between the charged groups on the polymer chains and Ca^++^ ions. The determination of the ionic content of gels unambiguously indicates that Ca^++^ (inside gel) > Ca^++^ (outside gel).

The influence of the addition of higher valent salts on the virial coefficients of highly charged polyelectrolytes evidently involves a subtle interplay of interactions. Measurements indicate that the addition of Ca salt to charged polyelectrolytes solutions with Na^+^ counter-ions makes the polymer chains considerably more flexible (i.e., the persistence length is reduced, often by a factor as large as 2 or more), a change in polymer conformational structure that should reduce the magnitude of repulsive excluded volume interactions [30,31,32]. Moreover, the multivalent ‘counter-ion cloud’ localized diffusely around the polyelectrolyte chains can be expected to more effectively ‘screen’ the bare charge of the polyelectrolyte chains involved in inter-polyelectrolyte interactions than the monovalent counter-ions [31,32]. The reduction in the strength of these predominantly *repulsive* interactions would then make hydrophobic, and other attractive interactions between the polymer segments more prevalent, leading naturally to a greater tendency towards polymer phase separation with the addition of Ca salt. However, these plausible arguments do not adequately explain the observed dependence of the polyelectrolyte virial coefficients. In particular, it has been reported that the size of large-scale supramolecular polyelectrolyte assemblies measured by static neutron scattering and dynamic light scattering in these solutions [30] is generally decreased with the addition of the Ca salt- an effect that one might expect from a decrease rather than an increase in the attractive interaction strength between the polyelectrolyte chains. Evidently, subtle many-body effects are involved in these solution property changes of polyelectrolyte solutions upon adding multivalent salts, and further experimental and simulation studies are needed to elucidate the nature of these interactions. Many-body interactions are inherent to the condensed state [27], and these interactions become especially prevalent in complex fluids where the separate pairwise potentials describing the van der Waals, charge and multipole long range interactions and the bond potentials of polymer species in the fluid, gives rise to a non-trivial coupling of these interactions. We may thus expect many-body effects to arise in simulations of complex fluids initially formulated in terms of a multiplicity of pairwise interaction potentials.

### 2.4. Scattering from Polymer Gels and the Estimation of the Osmotic Compressibility

The thermodynamic interactions between the polymer and solvent molecules govern the scattering response of polymer solutions and gels. In semi-dilute polymer solutions [46] the average spatial extent of the thermal concentration fluctuations is defined by a correlation length *ξ*, which is the result of short-range van der Waals interactions. The correlation length can be thought of as being an effective mesh size of the overlapping array of macromolecules and can be measured by small angle scattering techniques, such as small angle neutron scattering or small angle X-ray scattering. In the case of random thermal concentration fluctuations, this scattering response is given by an Ornstein-Zernike line-shape, [47]

(5) $$I_{\mathrm{os}}(q)=\Delta \rho^{2}\;\left(\frac{k_{B}T}{K_{os}}\right)\;{\left(1+q^{2}\xi^{2}\right)}^{-1}$$

where *q* is the transfer wave vector, ∆*ρ*^2^ is the contrast factor [48] between the polymer and solvent, *k_B_* is the Boltzmann constant, *T* is the absolute temperature, *K_os_* = *φ* ∂Π/∂*φ* is the osmotic compression modulus of the system. The amplitude of the scattering vector *q* is given by *q* = (4*π*/*λ*) sin(*θ*/2), where *λ* is the wavelength of the incident neutrons and *θ* is the scattering angle. The subscript os refers to thermodynamic (osmotic) concentration fluctuations.

For systems containing linear segments of length *L* the following expression [49] valid in the Guinier approximation *qξ* < 1, can be used to model the osmotic contribution to the scattering intensity,

(6) $$I_{\mathrm{os}}(q)=\Delta \rho^{2}\;\left(\frac{k_{B}T}{K_{os}}\right)\;{\left(1+\mathrm{qL}\right)}^{-1}\;{\left(1+q^{2}R^{2}\right)}^{-1}$$

The characteristic scale *R* is interpreted as the effective cross-sectional dimension of the polymer from a coarse-grained viewpoint. In polymer gels the description of the scattering response requires an additional term due to the presence of large-scale static structures. These features give rise to extra scattering at small *q*, often due to surface scattering, which takes the form of a power law,

*I*_x_(*q*) = *A q*^−*m*^ (7)

where *A* is a constant and *m* = 4 in the case of smooth surfaces (Porod scattering) [50]. Increasing the surface roughness is reflected in a lower value of *m* [51]. The total scattering intensity thus takes the form,

(8) $$I(q)=I_{\mathrm{os}}\;(q)+I_{s}(q)=\Delta \rho^{2}\;\left(\frac{k_{B}T}{M_{os}}\right)\;{\left(1+\mathrm{qL}\right)}^{-1}\;{\left(1+q^{2}R^{2}\right)}^{-1}\;+\;{Aq}^{-m}$$

The Ornstein-Zernike expression (first term) in Equation (8) corresponds to thermal fluctuations. *M_os_* = *φ* ∂*Π_sw_* / ∂*φ* + (4/3) *G_s_* is the longitudinal osmotic modulus, and *Π_sw_* is the swelling pressure of the gel. The second term in Equation (8) describes the contribution of static concentration fluctuations. The longitudinal osmotic modulus is a particularly important quantity because the scattering intensity arising from thermodynamic concentration fluctuations *I_os_*(*q*) is governed by *M_os_*.

### 2.5. Osmotic Pressure Estimates of Polyelectrolyte Gels and Virial Coefficients

**Figure 2:**
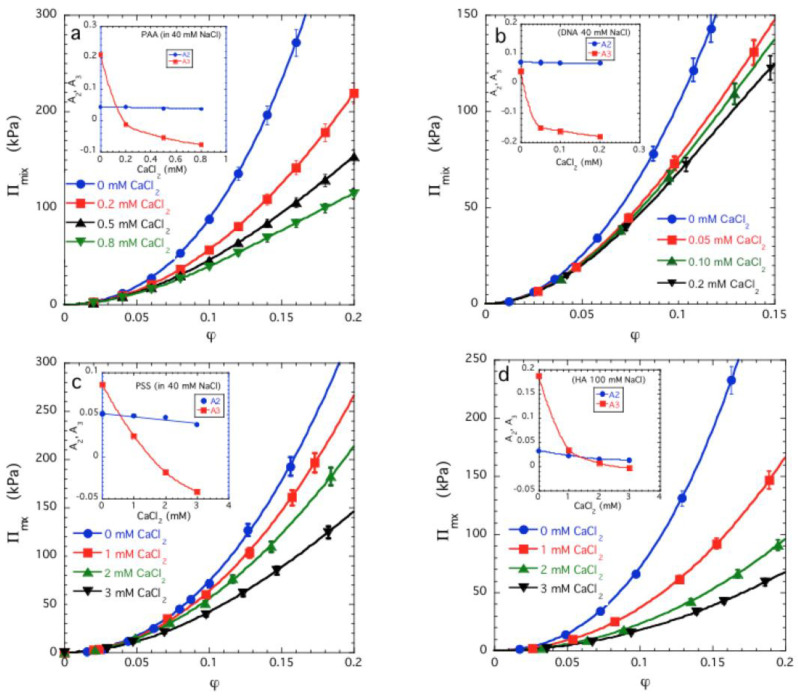
Mixing pressure *Π_mix_* versus polymer volume fraction *φ* plots for sodium polyacrylate (PAA) (**a**), DNA (**b**) and PSS gels (**c**) in 40 mM NaCl solution with different CaCl_2_ concentrations, and for HA gels (**d**) in 100 mM NaCl with different CaCl_2_ concentrations. The continuous lines show the fits of Equation (3) to the experimental data. Insets: Variation of *A*_2_ and *A*_3_ as a function of the CaCl_2_ concentration. The data uncertainties are estimated by one standard deviation of the linear regression fit parameters.

The dependence of *Π_mix_* as a function of *φ* is shown in Figure 2a for sodium polyacrylate (PAA) gels that have been swollen in 40 mM NaCl solutions containing different amounts of CaCl_2_ [33,34,35]. It can be seen that *Π_mix_* decreases with increasing concentration of divalent cations. The lines through the experimental data points are least squares fits to Equation (4). As shown in the inset, addition of divalent counter-ions causes a weak decrease in the value of *A*_2_ over the entire concentration range explored, while *A*_3_ strongly decreases as the divalent ion concentration increases and thereafter exhibits a slow decrease. Although some of these data were reported previously [33], but earlier observations were analyzed in terms of the Flory-Huggins interaction parameters *χ*_0_ and *χ*_1_ so the significance of those results in terms of the virial coefficients was not appreciated. The same situation holds for the other polyelectrolyte gels that we next consider. Figure 2b–d show similar data for DNA gels (Figure 2b [36]) and sodium poly(styrene sulfonate) gels (PSS) (Figure 2c) swollen in 40 mM NaCl solution, and for hyaluronic acid gels (HA) (Figure 2d) swollen in 100 mM NaCl solution with different CaCl_2_ contents. In all cases *Π_mix_* decreases with increasing Ca salt concentration. The variation of *A*_2_ and *A*_3_ with the CaCl_2_ concentration is similar in all four chemically different gels, indicating that all these systems exhibit an inverted variation in *A*_2_ and *A*_3_ from ‘ordinary gels’ where the onset of the deswelling transition is signaled by a change in the sign of *A*_2_ while *A*_3_ remains positive. The relative insensitivity of the observed trends to the specific chemical nature of the polyelectrolyte is also striking.

**Figure 3:**
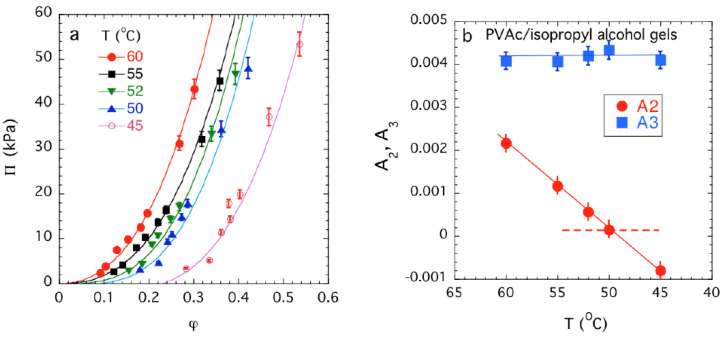
Mixing pressure *Π_mix_* versus polymer volume fraction φ plots for uncharged polyvinyl acetate (PVAc) gels [50]. The continuous lines show in (**a**) show fits of Equation (4) to the experimental data and the variation of *A*_2_ and *A*_3_ as a function of temperature are shown in (**b**). Note that *A*_3_ is small in magnitude and *A*_2_ changes sign as *T* varies, signaling the deswelling of the gel. The dotted line indicates *A*_2_ = 0, which defines the theta point condition [6,7]. The data uncertainties are estimated by one standard deviation of the linear regression fit parameters.

We contrast the observations made on model polyelectrolyte gels with the behavior of a model uncharged polyvinyl acetate (PVAc) gel swollen in isopropyl alcohol. In Figure 3, we varied the excluded volume interaction over an appreciable range by changing the temperature over a wide range in PVAc gels. We observed in this uncharged gel that *A*_2_ varies nearly linearly with temperature, while *A*_3_ remains small and positive. This is the typical behavior for the variation of the virials for simple fluids having short range interactions and no propensity towards molecular association [52]. When *A*_2_ becomes significantly attractive the gel de-swells as in the case of the polyelectrolyte gels with Ca salt added to alter ‘solvent quality’.

### 2.6. Scattering from Polymer Gels—An Alternative Approach to Estimating Osmotic Properties of Hydrogels

The estimation of the osmotic pressure of gels and polymer solutions is generally a labor-intensive and time-consuming process that requires meticulous care and correspondingly, such measurements covering a wide range of salt concentrations, temperature, etc., are not often reported. [See Materials and Methods section at the end of our paper, which describes measurement details.] Alternatively, information about the osmotic pressure, or its derivative, the osmotic compressibility, can be obtained from neutron and other scattering measurements, and this type of measurement also offers an opportunity of checking the self-consistency between osmotic pressure and osmotic compressibility measurements.

**Figure 4:**
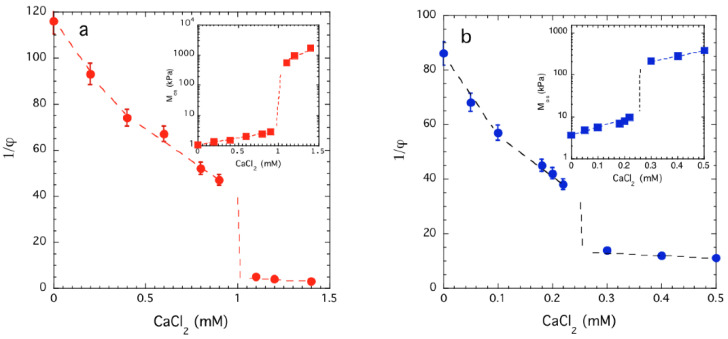
Dependence of the equilibrium swelling ratio (1/φ) of PAA (**a**) and DNA gel (**b**) in 40 mM NaCl as a function of the CaCl_2_ concentration. Insets: Variation of the longitudinal osmotic modulus *M_os_* calculated from Equation (9) on the CaCl_2_ concentration. Dashed lines are guide to the eyes. The uncertainties are the standard deviation based on three repeated measurements.

In what follows, we investigated the effect of the addition of Ca^++^ ions on the osmotic compressibility of model polyelectrolyte gels estimated from neutron scattering intensity measurements. In all these systems, the addition of CaCl_2_ gradually reduces the solvent quality and above a critical threshold concentration leads to a swelling-deswelling or ‘volume transition’, the manifestation of phase separation in a hydrogel. This striking phenomenon is illustrated in Figure 4, where the variation of the gel swelling degree (1/*φ*) is plotted as a function of the CaCl_2_ concentration in the equilibrium liquid for a PAA (Figure 4a) and a DNA (Figure 4b) gel. CaCl_2_ was added gradually to the solvent surrounding each gel, which had been previously allowed to swell in a 40 mM NaCl solution. At low Ca^++^ ion concentrations the gel volume progressively decreases, while at a critical concentration (in PAA at approximately 1 mM CaCl_2_ and in DNA at approximately 0.3 mM CaCl_2_) a sharp contraction of the gel specimen is observed. Although this type of volume transition has been observed before in many charged and uncharged hydrogels, there are several aspects of this ubiquitous transition that are not fully understood [53,54,55,56,57].

In systems undergoing a volume transition, it is expected that the thermodynamic changes affect the scattering response primarily the first term of Equation (8), through modifying *M_os_*, i.e., the amplitude of the dynamic component of the scattering intensity. Changes in the second term of Equation (8), however, mainly reflect the redistribution of the polymer within the gel at large length scales. The experimentally determined concentration dependence of *A*_2_ and *A*_3_ allows us to evaluate the polymer volume fraction at equilibrium swelling as a function of the CaCl_2_ concentration. Hence, the variation of the longitudinal osmotic modulus *M_os_* can be calculated from Equation (5):

*M_os_* = *φ* ∂Π_sw_/∂*φ* + (4/3) *G_s_* = *φ*^2^ (RT/V_1_) (2 *A*_2_
*φ* + 3 *A*_3_
*φ*^2^ + …) + (4/3) *G_s_* (9)

The insets in Figure 4a,b, show that a sudden change of *M_os_* occurs close to the CaCl_2_ concentration at which the volume transition is observed. According to Equation (8), the intensity scattered by thermodynamic concentration fluctuations is proportional to *φ*^2^/*M_os_*. This quantity can be calculated independently from macroscopic osmotic pressure measurements based on Equation (5), providing an important consistency check on the osmotic pressure and the scattering intensity measurements.

**Figure 5:**
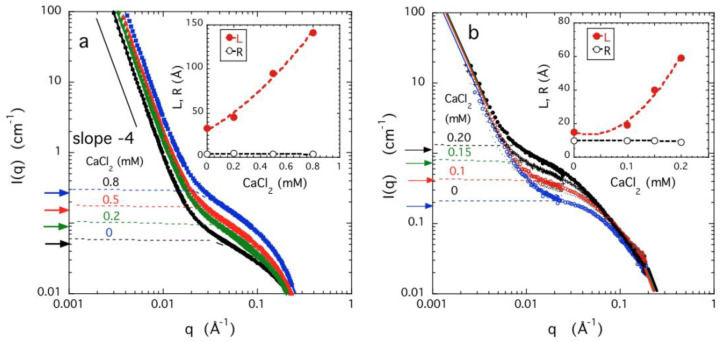
SANS profiles of PAA gels (**a**) and DNA gels (**b**) at c = 0.03 g/cm^3^ swollen in 40 mM NaCl solutions with different concentrations of CaCl_2_. Continuous lines are fits to Equation (8), dashed lines are the corresponding dynamic components [*I_os_*(*q*)]. Horizontal arrows at left axis are the values of *I_os_*(0) calculated from Equation (9) using the osmotic data of Figure 2a,b. Insets: dependence of the correlation length *L* and the cross-sectional radius *R* on the CaCl_2_ concentration in 40 mM NaCl concentration.

Figure 5a,b show the neutron scattering profiles of PAA and DNA gels as a function of the CaCl_2_ content. In all cases, a strong upturn is observed at small values of *q*. This upturn is characteristic of not only charged hydrogels, but this phenomenon is also observed rather generally in polyelectrolyte solutions where there is no chemical cross-linking. This rather universal low *q* excess scattering is another many-body effect [31,32] indicative of a general tendency towards supramolecular assembly. This type of clustering is essential to the analytic model of Dudowicz et al. [8,24], illustrating the virial sign inversion within an exactly solvable model.

The plateau region at higher *q* implies that the gels are homogeneous on intermediate length scales so these materials exhibit solution-like properties that may be described by the first term of Equation (8). The continuous line through the neutron data points is the least-squares fit to Equation (8). For these samples we conclude that a single static term together with a solution-like term satisfactorily describes the neutron scattering response.

Figure 5 also compares the dependence upon the calcium concentration of *φ*^2^/*M*_os_, calculated from macroscopic measurements (arrows directed to the *y*-axis) with the intensity of the dynamic scattering component measured by SANS (dashed curves). The latter increases with increasing CaCl_2_ concentration reflecting the reduction in the osmotic modulus as the volume transition is approached. The agreement between the SANS and osmotic results indicates that the effective molecular interactions that govern the volume transition are independent of the observational length scale; i.e., the large structures detected in the SANS experiment do not affect the thermodynamic properties of the gel.

### 2.7. Future Directions

We finally make some comments about future directions. Apart from the obvious need to better understand the factors (molecular parameters and general thermodynamic conditions) that control supramolecular assembly in polyelectrolyte solutions and gels, it is evidently important to understand even empirically how to control the sensitivity of polyelectrolyte solutions to higher valence counter-ions and salts that make these materials highly unstable for phase separation. This control is important in the context of natural polyelectrolyte gels such as aggrecan, which must be insensitive to variations of salt concentration for protecting joints from damage through everyday activities where these biological materials are subjected to large stresses. The formulation of successful drug therapies should conceivably be compromised by a strong sensitivity to counter-ion type and the presence of salts in the biological medium in which the drug is introduced.

As a final topic, we discuss some preliminary observations showing how the modification of a synthetic polyelectrolyte to have a bottlebrush structure, as in aggrecan and many other naturally occurring polyelectrolytes [58] alters the pattern of phase behavior observed in model polyelectrolyte gels discussed in the main body of this paper.

**Figure 6:**
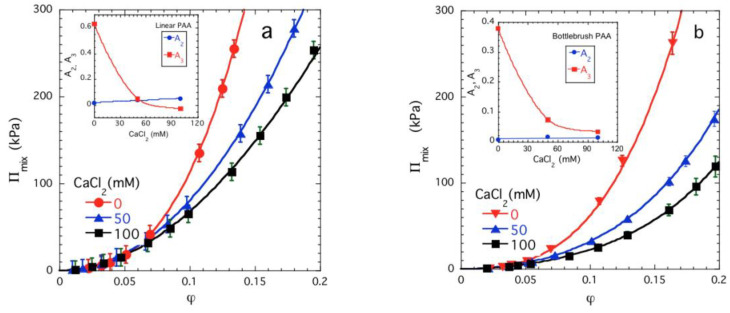
Mixing pressure *Π_mix_* versus polymer volume fraction φ plots for linear (**a**) and bottlebrush (**b**) PAA solutions in CaCl_2_ solutions. The continuous lines show fits of Equation (3) to the experimental data and the variation of *A*_2_ and *A*_3_ as a function of the CaCl_2_ concentration is shown in the insets. Note that *A*_2_ is very small and practically independent of the Ca salt concentration, *A*_3_ decreases with added Ca salt, but in the solution of the bottlebrush polymer it does not become negative. Accordingly, the latter solutions are relatively stable against phase separation in the form of a volume transition. The data uncertainties are estimated by one standard deviation of the linear regression fit parameters.

Figure 6 compares the results of exploratory osmotic pressure measurements made on model linear (Figure 6a) and bottlebrush (Figure 6b) PAA solutions as a function of added CaCl_2_ concentration. We observe that the grafting of the side chains appears to completely inhibit the sign change of *A*_3_ in the salt concentration range where the same polymer having a linear topology underwent a sign inversion with *A*_3_, and the usual volume transition with added salt is suppressed in this salt concentration range. Modifying the polymer topology has evidently induced a rather profound change on the miscibility pattern of the polyelectrolyte gel, and more importantly from an applications and medical science standpoint, this change in molecular topology has stabilized the polyelectrolyte gel against salt-induced structural ‘collapse’. This and other approaches to modifying the many-body interactions that underlie the supramolecular assembly and associated solvent quality trends deserve further systematic investigation.

We note that modulating the sensitivity of osmotic pressure to changes of salt, temperature, and factors influencing hydrogel stability also has profound consequences for controlling the fluid permeability and the rate of swelling and deswelling of gels when a change of thermodynamic conditions occurs. In particular, Tanaka and coworkers have demonstrated that the friction coefficient governing these rate processes is related to the solvent viscosity and the osmotic compressibility [59,60,61,62]. It would then appear that it should be possible to exert large changes in the permeability of polyelectrolyte gels composed of linear polymer chains through the introduction of relatively low concentrations of divalent salts, but that this sensitivity to salt should be greatly diminished if the polyelectrolyte had bottlebrush architecture. The type of salt insensitivity could have important implications for the functioning of the aggrecan molecules in joint tissue where the regulation of the rate of solvent flow is crucial to prevent tissue damage. The investigation of higher valence ions on fluid permeability and swelling of polyelectrolyte hydrogels is then a topic of obvious interest for biomedical applications.

In addition to variations of molecular topology, it would be of interest to investigate ‘block copolymer gels’ in which gels of different type are embedded in each other to obtain a composite material in which swelling of one block modules the mechanical properties of the gel material by a tensegrity mechanism. Articular cartilage, the load- bearing tissue of the joints of animals and humans can be viewed as such a material and many of the special mechanical properties of cartilage has been attributed to the composite nature of these natural gel materials. With this physical model of articular cartilage in view, Horkay and Basser [63] have recently produced a composite gel in which charged PAA microgel particles were dispersed in a continuous polyvinyl alcohol (PVA) polymer matrix. It was found that the load-bearing capacity of articular cartilage could be reproduced to a remarkable degree with this synthetic gel material. The osmotic and elastic properties of polyelectrolyte gels described in the present work provide essential insight into this novel type of gel having profound importance with respect to treating joint injuries and arthritis. The investigation of the swelling and elasticity of these gels represents an entirely new field of study.

## 3. Conclusions

We have confirmed a general tendency of polyelectrolyte hydrogels to exhibit a distinct pattern of miscibility from simple fluids and uncharged hydrogels. In particular, we observe that the second virial coefficient remains positive while the third virial coefficient changes sign with increasing Ca^+2^ ion concentration, ultimately leading to phase separation in terms of a swelling-deswelling volume transition of several model polyelectrolyte gels. Our findings are consistent with simulation studies of the hydrophobic effect in aqueous solutions and heuristic arguments by De Gennes for aqueous PEO polymer solutions where it was suggested that many-body interactions could give rise to this type of inversion of sign in the second and third virial coefficients. This phenomenon was later predicted in analytic calculations of complex fluids exhibiting cooperative self-assembly into dynamic polymeric structures by Dudowicz and coworkers [7,28] and our scattering observations further confirm that supramolecular assembly is a general feature of the polyelectrolyte gel materials studied in the present work. We infer from these observations that many-body interactions are generally important in polyelectrolyte materials. The associative interactions that give rise to significant supramolecular assembly in polyelectrolyte solutions [30] also give rise to a qualitative change in the thermodynamics of polymer miscibility and phase separation.

As a control study, we investigated uncharged (polyvinyl acetate) gels where solvent quality was conveniently varied by varying the temperature (salt is normally not effective in varying solvent quality in uncharged gels) where we found no inversion in the variation of the second and third virials near the onset of gel phase separation. The swelling-deswelling behavior in this gel follows the familiar pattern of simple fluids so that many-body interactions are evidently not as important in uncharged gel materials. We must remember that PEO is an uncharged polymer and it is certainly possible for many-body effects to become prevalent in even uncharged aqueous polymer hydrogels. This possibility deserves further study.

Since the characterization of charged and uncharged hydrogels and polymer solutions by osmotic pressure measurements is often arduous, we also checked whether similar thermodynamic information can be extracted from neutron scattering measurements of the osmotic compressibility. We find full consistency between the neutron scattering and direct osmotic measurements, providing a more accessible approach for obtaining information about the osmotic virial measurements. We expect this approach of estimating osmotic virial coefficients to become useful in the future. Most of the virial coefficient estimates reported in the present paper are based on osmotic pressure measurements that have been reported in the literature over the last two decades. This fact shows the value of transcribing already existing data for Flory *χ_i_*-interaction parameters into their equivalent virial coefficients [64]. This not only avoids ambiguities in these empirical interaction parameters, such as their reported concentration dependence, but allows for a discussion of this type of thermodynamic information in terms of more fundamental osmotic virial coefficients. Now that these interaction parameters are being systematically tabulated for materials design applications, it is recommended that databases should be made for the virial coefficients which can be more readily interpreted physically based on a statistical mechanical framework. It is only in this framework that the trends in the virials of charged hydrogels can be recognized as being qualitatively distinct from simple fluids. We expect that a similar mapping of *χ_i_*-interaction parameters to their virial counterparts will reveal new patterns of miscibility in polymer blends, block copolymer materials and polymer solutions. It has already been observed theoretically that radically different patterns of phase separation occur in polymer blends due to differences in the shape of the monomers of the component polymers, along with qualitatively different variations in the *χ*-interaction parameters with monomer structure [27,28], but the free energy of these blends becomes universal when represented in terms *A*_2_ and *A*_3_ [7]. As a final point, we note that tabulated virial coefficients should allow for the input of a more realistic description of the free energy density of the material in coarse-grained models of phase separation of complex fluids, which should allow these models to be more predictive [64,65]. Recently, it has become appreciated that many biological structures and disease states result from the phase separation of naturally occurring polyelectrolytes [66,67,68].

## 4. Materials and Methods

### 4.1. Preparation Poly(acrylic Acid) and Polystyrene Sulfonate Gels

Poly(acrylic acid) and polystyrene sulfonate gels were synthesized by free-radical copolymerization of the monomers (acrylic acid and styrene sulfonate) and the crosslinker (*N*,*N*′-methylenebis(acrylamide) in aqueous solution following the procedure described by Sugitani et al. [69] Special molds were used to make cylindrical (1 cm height, 1 cm diameter) gels. The monomer concentration was a mass fraction of 30%. In the initial mixture 35% of the monomers were neutralized by sodium hydroxide. Gels were prepared by copolymerization of aqueous solutions containing 30% (mass/mass) monomers and 0.04% (mass/mass) *N*,*N*′-methylene-bisacrylamide crosslinker. After the components were mixed, dissolved oxygen was eliminated by bubbling nitrogen through the solution. The polymerization reaction was initiated by ammonium persulfate (0.5 g/L). Gelation was achieved at 80 °C. The gel solutions were kept at 80 °C for 2 h and then were allowed to set at room temperature for 20 h to ensure that the reaction was complete. Gel cylinders were removed from the mold, neutralized fully, and placed in deionized water to remove any unreacted materials and other components (sol fraction, excess ions) not attached to the network. Water was replaced every day for 2 weeks. The swelling equilibrium concentration of the gels was determined in pure deionized water and in solutions of different salts. For the SANS experiments, gels were made using the same procedure in D_2_O in the form of slabs to fit the sample holder.

**Scheme 1:**
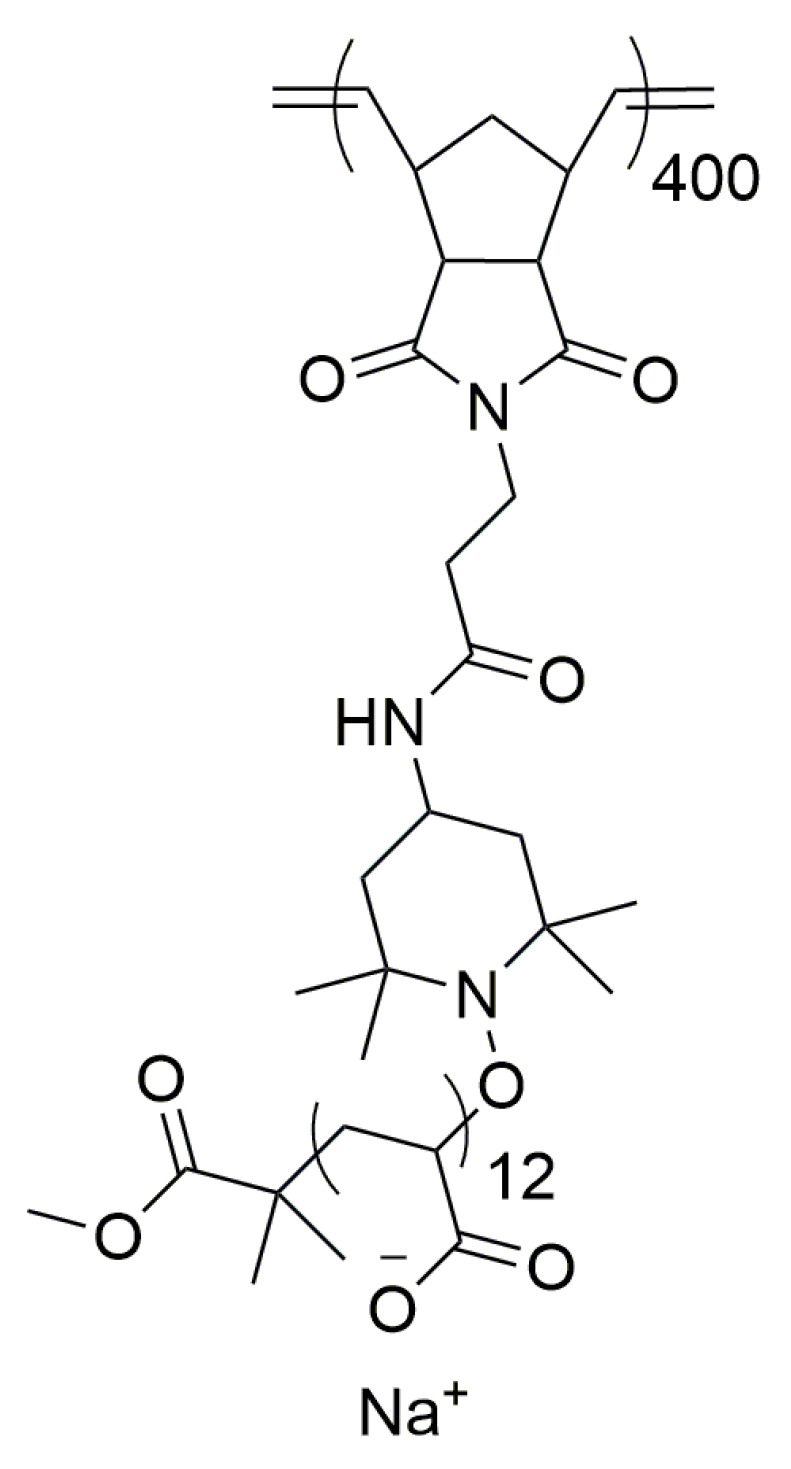
Structure of the monomer of bottlebrush PAA polyelectrolyte [70].

Bottlebrush PAA was made by a procedure described previously (Scheme 1) [70,71,72]. The polymer used in this study had a number average relative molecular mass, *M*_n_ = 678 kDa, dispersity: Đ = 1.13 with the degree of polymerization 400 and 12 for the backbone and side chain, respectively.

### 4.2. Preparation of DNA and HA Gels

DNA gels were made from deoxyribonucleic acid sodium salt (Na-DNA from salmon testes, Sigma, St. Louis, MO, USA) [73]. According to the manufacturer the % G-C content of this DNA is 41.2%, and the melting temperature is reported to be 87.5 °C in 0.15 M sodium chloride plus 0.015 M sodium citrate. The molecular mass determined by ultracentrifugation is 1.3 × 10^6^, which corresponds to approximately 2000 base pairs.

First, DNA was dissolved in 4-(2-hydroxyethyl)-1-piperazine ethanesulfonic acid (HEPES) buffer (pH = 7.0) then the solutions were dialyzed against distilled water. DNA gels were made by cross-linking [73] with ethyleneglycol diglycidyl ether (2%) at pH = 9.0 using tetramethylethylenediamine (TEMED) to adjust the pH. The DNA concentration at cross-linking was 3 mass %. The gels were equilibrated in NaCl solutions containing different amounts of CaCl_2_ (0 mM to 0.2 mM). For the SANS experiments, DNA gels were made in D_2_O in the form of slabs adjusted to fit the sample holder.

HA gels were made of solutions of sodium hyaluronate (HA, mass average relative molecular mass *M_w_* = 1.2 × 10^6^, dispersity: Đ = 1.5) prepared in H_2_O. The samples were allowed to homogenize for 2 d to 3 d before cross-linking. HA solutions (3 mass %) were cross-linked with ethylene glycol diglycidyl ether (Fluka) at pH = 9.0 using TEMED to adjust the pH. Isometric cylindrical samples of 1 cm height were made in a mold for uniaxial compression measurements. The gels were equilibrated either with H_2_O or with salt solutions.

### 4.3. Preparation of Poly(vinyl Acetate) Gels

Aqueous polyvinyl alcohol solutions of different concentrations (c = 3.0, 6.0, 9.0 and 12.0) mass % were cross-linked with glutaraldehyde (GDA, Merck) at pH = 1.5 and *T* = (298 ± 0.1) K [74]. Networks with different cross-linking densities were prepared. The cross-linking density (mole fraction of the cross-linking agent (GDA) in the dry network, was varied from 2 × 10^−3^ to 2 × 10^−2^ (for the calculation of the moles of monomer, units of PVA were used).

Cylindrical network samples 1 cm in diameter and 1 cm in height were made in a suitable mold. The mixture of polymer, cross-linker, and catalyst (2 N HCl solution) was stirred by a magnetic stirrer and then poured into the mold. After the cross-linking reaction gels were removed from the mold and unreacted polymeric materials and HCl were removed by distilled water. Then, the media of the gels was replaced by a mixture of acetic anhydride (40 vol %)-acetic acid (10 vol %)-pyridine (50 vol %). The acetylation reaction was performed at 363 K for 8 h. During this process, the acetylation mixture was renewed hourly. In the last 3 h the acetic acid was omitted from the mixture, in order to shift the equilibrium to the direction of acetate formation. The samples were washed with toluene. The complete wash cycle involved not less than 10 solvent exchanges and took over 1 month. Then, the gels were dried. The extent of acetylation was measured and an agreement within 1% to 2% was found between the calculated and experimentally determined values. The dry networks were swollen in isopropyl alcohol. The gels were kept 1 month at each *T* before testing.

### 4.4. Osmotic Swelling Pressure Measurements

Hydrogels were equilibrated with poly(vinyl pyrrolidone) (PVP) solutions (molecular mass: 29 kDa) of known osmotic pressure [75,76] A semipermeable membrane was used to prevent penetration of the polymer into the network. Periodically the mass of the gel (m_g_) was measured, and this value used to calculate the total polymer weight fraction. Equilibrium was achieved when no further changes in either gel swelling degree or solution composition were detectable. To reach equilibrium took 8 d to 10 d, depending on the size of the swollen gel. At this point, gel samples were removed from the dialysis bags, weighed, and dried. The volume fractions were calculated from the known densities of the polymers and the solvent. The gel samples were dried at 95 °C. Reversibility was checked by transferring the gels into PVP solutions at different osmotic pressure values. This procedure gives for each gel the dependence of *Π_sw_* upon polymer volume fraction *φ*. When deswelling was carried out in salt solutions, it was assumed that the salt concentration in the surrounding liquid phase did not change appreciably during swelling; i.e., the amount of equilibrium solution is sufficiently large (infinite bath).

**Scheme 2:**
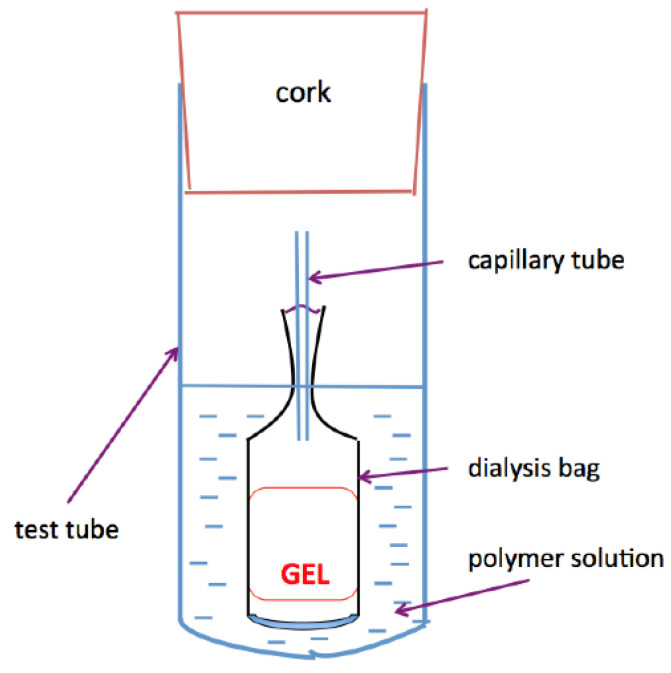
Apparatus to measure the osmotic swelling pressure of polymer gels.

Scheme 2 shows the apparatus designed to perform osmotic swelling pressure measurements. The glass capillary tube in the dialysis bag ensures that the pressure inside the bag is equal to that in the environment. Swelling pressure measurements were made on at least 3 nominally identical gel samples at (25 ± 0.1) °C. Repeated measurements showed a mean change in the osmotic swelling pressure less than 5%.

### 4.5. Shear Modulus Measurements

The shear modulus of the gels was determined using a TA.XT2I HR Texture Analyser (Stable Micro Systems, Godalming, UK); See disclaimer statement. The measurements were performed under uniaxial compression on cylindrical specimens equilibrated with salt solutions. Typical sample sizes were (0.5 to 1) cm in height and (0.5 to 1) cm in diameter. The shear modulus, G_s_, was calculated from the nominal stress, σ (force per unit undeformed cross-section), using the relation, [38].

σ = G_s_ (*Λ* − *Λ*^−2^) (10)

where *Λ* (= *L*/*L_o_*, *L* and *L_o_* are the lengths of the deformed and undeformed gel specimen, respectively) is the deformation ratio.

Uniaxial compression measurements were made in the range 0.7 < *Λ* < 1. The absence of volume change and barrel distortion during these measurements was checked (Typical duration of a stress–strain measurement was between 5 min and 10 min). Both osmotic and mechanical measurements were carried out at (25 ± 0.1) °C.

### 4.6. Small-Angle Neutron Scattering

SANS measurements were made at the National Institute of Standards and Technology (NIST), Gaithersburg, MD, on the NG3 instrument with incident wavelength of 8 Å. The sample-detector distances used were 3 m and 13.1 m, corresponding to an explored wave vector range 0.003 Å^−1^ ≤ *q* ≤ 0.15 Å^−1^. The ambient temperature during the experiments was (25 ± 0.1) °C. The gel samples were prepared in solutions of heavy water. Standard 2 mm NIST sample cells were used. After radial averaging, corrections for incoherent background, detector response, and cell window scattering were applied. The neutron scattering intensities were calibrated using absolute intensity standards available at NIST [77]. 

## Data Availability

The data that support the findings of this study are available from the corresponding authors upon reasonable request.
